# The mental health of preschoolers in a Norwegian population-based study when their parents have symptoms of borderline, antisocial, and narcissistic personality disorders: at the mercy of unpredictability

**DOI:** 10.1186/1753-2000-6-19

**Published:** 2012-05-20

**Authors:** Turid Suzanne Berg-Nielsen, Lars Wichström

**Affiliations:** 1 Regional Centre for Child and Adolescent Mental Health, Faculty of Medicine, Norwegian University of Science and Technology, Trondheim, Norway; 2 Department of Psychology, Norwegian University of Science and Technology, Trondheim, Norway; 3 NTNU Social Science, Trondheim, Norway; 4 Child and Adolescent Psychiatric Clinic, St. Olav’s Hospital, Trondheim University Hospital, Trondheim, Norway

**Keywords:** Personality disorder, Psychiatry, Psychopathology, Child, Parent, Generation

## Abstract

**Background:**

Clinical studies have shown that children of parents with mental health problems are most likely to develop psychiatric problems themselves when their parents have a Personality Disorder characterized by hostility. The Personality Disorders that appear most associated with hostility, with the potential to affect children, are Borderline Personality Disorder, Antisocial Personality Disorder and Narcissistic Personality Disorder. The question addressed in this study is whether the risk to children’s mental health extends to the normal population of parents who have subclinical symptomlevels of these disorders.

**Methods:**

This inquiry used data from a Trondheim, Norway community sample of 922 preschoolers and one parent for each child. The mean age of the children was 53 months (SD 2.1). Parents reported symptoms of Borderline, Antisocial and Narcissistic Personality Disorders on the DSM-IV ICD-10 Personality Questionnaire, and the children’s symptoms of DSM-IV behavioral and emotional diagnoses were measured with the Preschool Age Psychiatric Assessment, a comprehensive interview. Multigroup Structural Equation Modeling was used to assess the effect of parents’ symptoms on their preschoolers’ behavioral and emotional problems.

**Results:**

The analyses yielded strongly significant values for the effect of parents’ Personality Disorder symptoms on child problems, explaining 13.2% of the variance of the children’s behavioral symptoms and 2.9% of the variance of internalizing symptoms. Biological parents’ cohabitation status, i.e., whether they were living together, emerged as a strong moderator on the associations between parental variables and child emotional symptoms; when parents were not cohabiting, the variance of the children’s emotional problems explained by the parents’ Personality Disorder symptoms increased from 2.9% to 19.1%.

**Conclusions:**

For the first time, it is documented that parents’ self-reported symptoms of Borderline, Antisocial, and Narcissistic Personality Disorders at a predominantly subclinical level had a strong effect on their children’s psychiatric symptoms, especially when the biological parents were not living together. Child service providers need to be aware of these specific symptoms of parental Personality Disorders, which may represent a possible risk to children.

## Background

Based on research conducted in Scandinavia, the National Institute for Public Health in Norway estimated that the prevalence of mental disorders among mothers is 28.9% and fathers 14.1% [1-3]. Mental disorders are also transmitted to the next generation; extensive research has documented that the children of parents with psychiatric disorders have a heightened risk of developing psychiatric problems themselves [4,5].

However, studies are limited on the possible transmission of emotional and behavioral problems to the children of parents with Personality Disorders (PD). This is the case even though M. Rutter and D. Quinton in their classic study from 1984 showed that children of parents with PDs characterized by hostility were more likely to develop mental health problems themselves compared with children of parents with severe psychiatric diagnoses like Schizophrenia or Bipolar Disorder or any other psychiatric diagnosis [6]. The PDs that appear to be most strongly associated with hostile behavior and that may affect children are Borderline Personality Disorder (BPD), Antisocial Personality Disorder (ASPD) and Narcissistic Personality Disorder (NPD) [7]. These disorders are characterized by features such as difficulty controlling anger (BPD, ASPD, NPD), impulsive and aggressive outbursts (BPD, ASPD), rage when being criticized (NPD), irritability (BPD), aggressiveness and physical assault (ASPD), being tough-minded, exploitive, and non-empathic (ASPD, NPD), lack of reciprocal interest and sensitivity to the wants and needs of others (ASPD, NPD), extreme sarcasm (BPD), being indifferent to having hurt another (ASPD), sudden and dramatic shifts in their view of others (BPD), emotional coldness (NPD, ASPD) and disdainful, arrogant behavior (NPD) [8,9]. A recent factor analytic study of BPD reveals three main factors that characterize the disorder: affect dysregulation (e.g., inappropriate anger); behavioral dysregulation and disturbed relations; and additional personality features, such as low conscientiousness and low agreeableness [10]. Interestingly, the disturbed and unstable relations factors are also associated with thought disorders [11], indicating flaws in the perception and interpretation of social relations. This latter factor may be especially relevant to how parents with BPD relate to their children. Obviously BPD, ASPD, and NPD have not only impulsive aggressiveness in common but also a pervading preoccupation with one’s own needs rather than those of others. Furthermore, they have little insight into the fact that their PD symptoms may be causing distress for others [12]. They also tend to misinterpret social interactions and readily attribute hostile intentions to others [13]. These traits may conceivably affect children just as much as impulsive aggressiveness.

More than ten years passed after Rutter & Quinton’s study before the next study documented that children of mothers with BPD were at risk for developing not only attention and disruptive behavior disorders but also a wide range of other psychiatric disorders [14]. The non-specificity of disorders being transmitted from BPD parents to the next generation is consistent with factor analytic studies of BPD showing associations in adults with both internalizing and externalizing dimensions [15]. Another ten years passed until the next major study of this topic, a study in which children of mothers with BPD were compared with children of mothers with depressive disorders, other personality disorders, or no psychiatric conditions [16]. The offspring of BPD mothers showed more problems than children of mothers with any other PDs or depression. Particularly notable was the children’s report of very low self-esteem.

Parents with comorbid diagnoses that include BPD are also more likely to have children with mental health problems; e.g., children of parents with Major Depressive Disorder (MDD) and BPD are 6.8 times more likely to exhibit a current or past diagnosis of MDD than children of parents with MDD but no BPD [17]. Another study showed that 23.7% of infants suffering from Non-Organic Failure to Thrive had parents with PDs [18]. Studies of children with parents with ASPD are rare because of the parents’ often uncooperative attitude toward service providers and investigators, yet the few studies available in the literature show that the offspring of parents with ASPD, like children of those with BPD, exhibit a wide range of psychiatric disorders, both internalizing and externalizing [19-22].

Genetic factors contribute approximately 40-50% of the variation in the development of personality disorders but it is unknown to what extent children with mental health problems whose parents have PDs will themselves develop PDs as adults. Behavioral geneticists have recently stressed the importance of non-shared environmental effects on the development of child personality and psychopathology [23,24]. Accordingly, the non-genetic association between parental BPD, ASPD and NPD and child psychopathology could conceivably be partially due to key features of the parents PDs differentially affecting offspring siblings. In one study, depressed mothers showed less investment in what they perceived to be a high-risk infant than in low-risk children in the family [25]. Parental erratic and unpredictable behavior might be disproportionally directed to some children in the family, however, to date, no known studies have examined differential parenting in parents with PDs.

### Personality disorders

Personality disorders are described in the Diagnostic and Statistical Manual of Mental Disorders (fourth edition) (DSM-IV) as inflexible and pervasive, with symptoms that have caused and continue to cause significant distress or negative consequences in interpersonal functioning as well as other aspects of life such as thoughts, feelings and impulse control [9]. The prevalence of BPD in the general United States population is estimated to be 5.9% (99% CI = 5.4 to 6.4) [26]. In contrast to previous findings in clinical populations, there is no difference between the male and female BPD rates. The overall ASPD prevalence in the same US sample is approximately 3.6%, and men are significantly more likely to have the disorder than women [27]. The prevalence of NPD in the same population is 7.7% for men and 4.8% for women [28].

An ongoing debate among researchers is whether PDs are continuously distributed dimensions or discrete categories, i.e., whether the difference in PD symptoms between population-based and clinical samples is mainly quantitative or if there is a qualitative difference between those who fulfill the PD diagnostic criteria and those who do not [12,29,30]. Recently, a continuous dimensional scoring of PDs has been proposed for inclusion in the DSM-5 [31].

### Persons with BPD, ASPD and NPD and parenting

The association between parents’ and offspring’s psychiatric disorders has been shown to be mediated partially by maladaptive parenting behavior [32-35]. Several studies exist on ‘normal’ personality traits as determinants of parenting [35,36] and of children’s problems [37,38]. However, less evidence is available on the effects of parental PD on parenting. This stands in contrast to several studies focusing on parental depression and parenting [39]. Yet, researchers have found that parents with PDs, especially mothers with BPD, clearly show dysfunctional parenting, even more dysfunctional, according to some studies, than parents with other psychiatric disorders [40-42]. In a community-based study, parents with PDs were three times more likely to engage in problematic child rearing behavior, with BPD and ASPD parents having particularly high rates of such behavior [43]. In a study of mothers involved in child care proceedings, 70% had a diagnosis of PD [44], and in a sample of mothers with Münchausen Syndrome by Proxy, 66% had BPD [45]. Mothers with a history of ASPD demonstrate unresponsiveness in interacting with their children [46], whereas mothers with BPD appear more ‘insensitively intrusive’ [47], with disrupted affective communication [48]. Fathers with ASPD are also especially hostile toward their sons [22,35,49]. In DSM-IV, parents with ASPD are described as individuals who may beat or neglect to care for their child in a way that puts the child in danger [9], and studies confirm that parents with a history of ASPD are at increased risk of abusing their children [50].

### Persons with BPD, ASPD and NPD as spouses

Individuals with ASPD tend to be irritable and aggressive and may repeatedly commit acts of physical assault, including spouse beating or child beating [9]. They may be irresponsible and exploitive in their sexual relationships and have a history of many partners, never sustaining a monogamous relationship. Individuals with BPD present angry disruptions in their close relationships and frequently ‘express inappropriate, intense anger’ [9]. This not only affects their children but also their partners; hence, their relationships are markedly unstable. Not surprisingly, parents with symptoms of BPD, ASPD and NPD often break up with their spouses and have to cope with single parenthood. Previous research has shown that the prevalence of psychiatric disorders among preschoolers is doubled when their biological parents do not live together [51]; hence, in studies of intergenerational transmission of psychopathology, the inclusion of parental cohabitation status is highly relevant.

Given that the children of parents with hostile and self-preoccupied PDs are more likely to develop psychiatric problems than children of parents with any other psychiatric diagnoses [6], an imminent question is whether this risk to children extends to the normal population of parents in whom PDs are predominantly at a subclinical level. Using a preschool sample for such a study could yield information on how early child problems might manifest that are associated with parental PDs.

### Research questions and hypotheses

1. We hypothesized a common latent factor underlying parents’ observed or “manifest” BPD, ASPD, and NPD symptoms called “Self-preoccupation and impulsive aggressiveness”. A latent factor or variable refers to the systematic variation linking a set of observed variables; latent variables represent what a set of observed variables have in common and they are less prone to contamination and unsystematic sources of variation such as measurement error [12,52].

2. We hypothesized a common latent factor for the children’s Attention-Deficit/Hyperactive Disorder (ADHD); Oppositional Defiant Disorder (ODD); and Conduct Disorder (CD) called “Externalizing”.

3. We hypothesized a common latent factor for the children’s symptoms of Major Depressive Disorder (MDD); Dysthymia Disorder (DyD); Generalized Anxiety Disorder (GAD); and Separation Anxiety Disorder (SAD) called “Internalizing”.

4. Given that in high-risk samples children of parents with BPD, ASPD, and NPD show a considerably increased risk of mental health problems, we hypothesized that a milder, yet significant, increased risk would be seen in families from a community sample. Using data from a population-based sample with Structural Equation Modeling, we wished to determine whether the latent parent factor Self-preoccupation and impulsive aggressiveness was a predictor of the latent child factors of Externalizing and Internalizing.

**Figure 1:**
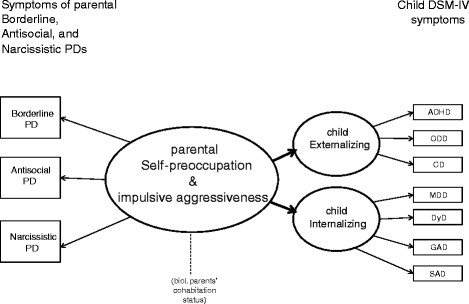
Hypothesized model.

The hypothesized associations between the parents’ and children’s observed symptoms and the relationships of the three latent variables are presented in Figure 1. Rectangles represent observed variables, ellipses represent the latent variables, and lines with single arrows represent the hypothesized direction of prediction.

5. Because the risk to a child’s mental health is doubled when the biological parents are not cohabiting and because this risk is already evident when a child is in preschool [51], the question was raised whether non-cohabitation of biological parents with BPD, ASPD, and NPD symptoms also increases the risk of behavioral or emotional problems in their preschoolers.

Cohabitation is defined as biological parents living together at the time of the study and for more than six months prior to the study.

## Methods

### Participants

A total of 922 parent–child pairs participated (73.8% of those who were initially invited). Only one parent was required to attend. Of the 922 parents, 920 had custody of the target child and were living with the child. In all, 902 parents were the child’s biological parents, 11 were adoptive parents, three were foster parents, two were stepparents, and one was a grandparent. Six parents provided information twice on sibling-pairs or twins (i.e., in six instances, two children from the same family participated in the study). The mean age of the children was 53.0 months (range 46.3 to 63.0, SD = 2.1).

The sample (adjusted for stratification) was compared with data from all parents of 4-year-olds in Trondheim in the years 2007 and 2008 using register information from Statistics Norway. While the sample contained significantly more divorced parents (6.8%) than the general population (2.1%), the education level was the same in both groups. The descriptive information about the sample is shown in Table 1. Several key indicators of the population of Trondheim are similar to those of the entire nation; e.g., the average gross income per inhabitant is 99.5% of the national average, the employment rate is identical to the national rate, and 80.0% of the households are two-parent families compared with a national average of 81.4% [53].

**Table 1:** Sample characteristics

**Characteristic**		**%**
Gender of child	Male	49.1
	Female	50.9
Gender of parent informant	Male	15.2
	Female	84.8
Ethnic origin of biological mother	Norwegian	93.0
	Western Countries	2.7
	Other countries	4.3
Ethnic origin of biological father	Norwegian	91.0
	Western Countries	5.8
	Other countries	3.2
Biological parents’ marital status	Married	56.3
	Cohabiting > 6 months	32.6
	Separated	1.7
	Divorced	6.8
	Widowed	.2
	Cohabiting < 6 months	1.1
	Never lived together	1.3
Informant parent’s socio-economic status	Leader	5.7
	Professional, higher level	25.7
	Professional, lower level	39.0
	Formally skilled worker	26.0
	Farmer/fisherman	.5
	Unskilled worker	3.1
Parent’s highest completed education	Not completed junior high school	0
	Junior high school (10^th^ grade)	.6
	Some educ. after jun. high school	6.1
	Senior high school (13^th^ grade)	17.3
	Some educ. after sen. high school	3.4
	Some college or university educ.	7.6
	Bachelor degree	6.2
	College degree (3–4 years study)	33.6
	Master’s degree or similar	20.3
	Ph.D. completed or ongoing	4.4
Households’ gross annual income	0 - 40,000 USD	3.3
	40,000 - 94,000 USD	18.4
	94,000 - 161,000 USD	51.6
	161,000 USD +	26.7
At least one parent had received treatment for mental health problems	None	73.8
	Outpatient only	16.3
	Hospitalized	10.0
Parents had received medical treatment for mental health problems	No	87.4
	Yes	12.6

### Recruitment

**Figure 2:**
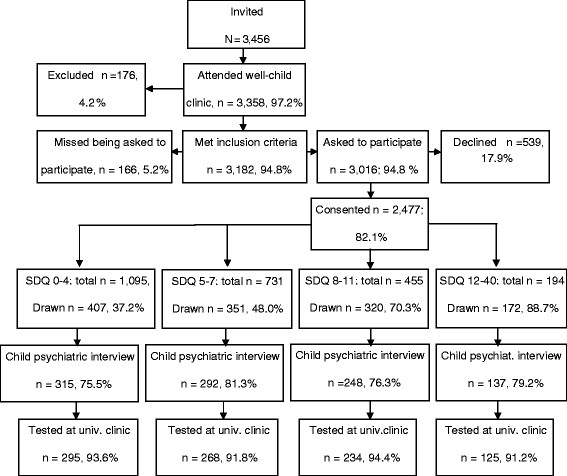
Sample recruitment.

The current study is part of the larger longitudinal “Trondheim Early Secure Study” (TESS) on mental health risks and protective factors in children. All children born in 2003 and 2004 and their parents in the city of Trondheim, Norway, were invited to participate. A letter of invitation together with the Strengths and Difficulties Questionnaire (SDQ, 4–16 version) [54] were sent to their homes. The parents brought in the completed SDQ when attending their scheduled appointment for the ordinary community health checkup for 4-year-olds. A flow chart describing the recruitment procedure and the participation rates is presented in Figure 2. Almost everyone who was eligible for the study appeared at the city’s well-child clinics, meaning that the sample was, in practice, a community sample. The parents whose Norwegian proficiency was insufficient to allow them to complete the SDQ were excluded (n = 176). The nurse at the well-child clinic informed the parents about the study and obtained their written consent to participate (5.2% of eligible parents were missed being asked). The study with all procedures and instruments were approved by the Regional Committee for Medical and Health Research Ethics.

The SDQ, which included 20 items yielding a total difficulties score, was used for screening [55]. The SDQ total difficulties scores were divided into four strata (cutoffs: 0–4, 5–8, 9–11, 12–40). Using a random number generator, a defined proportion of parents in each stratum was selected to participate in a structured diagnostic interview concerning their children’s mental health. The selection probabilities increased with increasing SDQ scores (the probabilities were .37, .48, .70, and .89 for the first, second, third, and fourth strata, respectively). Of the 1,250 parents who were invited to participate, we were able to test and interview 922 (73.8%). One parent was interviewed and attended further sessions at the university clinic with the child. Of the 922 parents 13 did not give information on their cohabitation status and thus were not included in some analyses. The dropout rate after consenting at the well-child clinic was not different across the four SDQ-strata (Chi-sq. = 5.70, df = 3, NS) or by gender (Chi-sq. = .23, df = 1, NS).

### Instruments

#### Psychiatric diagnostic interview: The preschool age psychiatric assessment (PAPA)

The PAPA [56] was conducted with one parent either at the University Clinic or in the family’s home some weeks after the visit at the well-child clinics. The PAPA is a semi-structured psychiatric interview for parents of children ages two through five years. The PAPA is based on the parent version of the Child and Adolescent Psychiatric Assessment (CAPA) for 9- to 18-year-olds. The PAPA assesses all of the DSM-IV-TR and ICD-10 criteria for 13 psychiatric diagnoses that are relevant to this age group. However, it does not attempt to diagnose autism spectrum disorders [56].

The PAPA uses a structured protocol involving both required questions and optional follow-up questions. The task of the interviewer is to ensure that the interviewee understands the questions and that she or he provides clear information concerning the symptom in question. Interviewers continue to probe until there is enough information to decide whether the symptom is present at pre-specified levels of severity. If so, its onset date is recorded along with its frequency of occurrence, when relevant. A three-month primary period was used, and diagnoses were generated by computer algorithms implementing the DSM-IV criteria [9].

Interviewers (n = 7) had at least a bachelor’s degree in relevant fields and extensive prior experience working with children and families. They were trained by the team who developed the PAPA. Regular meetings with master coders were held, and the interviewers were observed from behind one-way mirrors to ensure adherence to the interview guide and avoid rater drift. Blinded raters recoded 9% of the interviewed audio recordings.

Because our sample was a low-risk, population based sample, we used the dimensional scores of seven of the most frequent diagnoses: ADHD, ODD, CD, MDD, DyD, GAD, and SAD. The multivariate interrater reliabilities between pairs of raters were measured using intra-class correlations (ICC): ADHD = .96; ODD = .97; CD = .91; MDD = .90, DyD = .93, GAD = .93; and SAD = .90.

#### The DSM IV and ICD-10 personality questionnaire (DIP-Q)

The DIP-Q was completed by one parent at the University Clinic some weeks after the diagnostic interview (PAPA). The DIP-Q is a 140-item true/false self-reported questionnaire yielding categorical diagnoses as well as dimensional measures of all ten DSM-IV and all eight ICD-10 personality disorders. The DIP-Q was developed based on the self-report screening instrument of the Structured Clinical Interview for DSM III-R Personality Disorders (SCID-II) [57] and analyses of the ICD-10 and DSM-IV diagnostic criteria [58-62]. Although somewhat over-inclusive compared with a structured expert clinical interview, validation studies have shown the DIP-Q to be a reliable screening instrument for PD symptoms and disorders in both psychiatric and non-clinical samples [58,60,62]. The Dip-Q has been used in several studies in Scandinavia; in the current study, only the DSM-IV (not ICD-10) Borderline, Antisocial, and Narcissistic PDs were dimensionally analyzed. A Yes/No answer format is applied in the Dip-Q, which violates the assumption of linearity that is required in the common measures of a measurement scale’s internal consistency, such as the Cronbach’s alpha. Therefore, a reliability coefficient applicable to categorical data, Theta, was used [63]. This procedure showed that the reliability for the different DIP-Q PDs varied between Θ = 0.71 and Θ =0.92.

### Statistics

Because we had a screen-stratified sample, to arrive at the correct estimations for the population, we conducted weighted analyses using weights proportional to the inverse of the probability of being selected as a subject (i.e., low screen scorers were “weighted up” and high scorers were “weighted down”). The Huber-White sandwich estimator was used to provide robust confidence intervals for the population [64,65].

Multigroup Structural Equation Modeling with latent variables was conducted using Mplus 6.1 [66], with a robust maximum likelihood estimator (MLR). The model’s goodness-of-fit was evaluated using the *χ*^2^ goodness-of-fit, the Comparative Fit Index (CFI), the Tucker-Lewis Index (TLI), and the root mean square error of approximation (RMSEA). Good model fit was defined by TLI ≥0.95, CFI ≥0.95, and RMSEA ≤ .05 [67]. Because the Chi-P is especially sensitive to sample size, with large samples other fit indices should be more decisive [52]; furthermore, the goodness-of-fit parameter estimates should also be considered when determining the model’s fit [68].

The parental “cohabitation status” variable was categorical. It was defined as the *biological* parents living together at the time of the study and for more than six months prior to the study. The information was obtained during the PAPA interview; however, interviewers missed asking 13 of the 922 participating parents about their cohabitation status.

## Results

### Observed variables in the model

The parent variables of BPD, ASPD and NPD, including the number and percentage of parents reporting 1–5 or more symptoms are presented in Table 2. A BPD diagnosis requires five or more symptoms of BPD, an ASPD diagnosis requires three or more symptoms of ASPD and a NPD diagnosis requires five or more NPD symptoms [9].

**Table 2:** Mean(SD) and number of PD symptoms and percentage of parents with 1–5 or more symptoms

	**All parents (=922)**			**Cohabiting parents (n = 785)**			**Non-cohabit. parents (n = 124)**		
	**BPD**	**ASPD**	**NPD**	**BPD**	**ASPD**	**NPD**	**BPD**	**ASPD**	**NPD**
Mean (SD)	1.38 (.05)	.66 (.02)	1.75 (.03)	1.29 (.05)	.64 (.02)	1.74 (.03)	1.95 (.19)	.81 (.08)	1.81 (.09)
# symptoms	%	%	%	%	%	%	%	%	%
0	47.8	50.1	.2	50.5	49.3	.2	45.1	46.2	0
1	15	37.4	52.8	15.3	36.6	51.5	8.6	33.8	54.3
2	17.6	10.0	26.6	16.8	8.6	26.4	18.4	18.1	20.9
3	7.9	1.8	14.7	7.5	1.8	12.9	7.9	1.4	21.1
4	4.8	.6	4.6	4.1	.6	4.8	7.9	.5	2.7
5	2.3	.1	.7	2.2	.1	.7	2.7	0	0
>5	4.6	.1	.1	3.8	.1	.5	5.2	0	.1
Diagnosis	≥5 sym.	≥3 sym.	≥5 sym.	≥5 sym.	≥3 sym.	≥5 sym.	≥5 sym.	≥3 sym.	≥5 sym.
	6.9%	.8%	.5%	6%	.8%	1.2%	9.4%	2%	0.1%

The mean of number of child symptoms and the percentages of children meeting the criteria for diagnoses are presented in Table 3.

**Table 3:** Mean number of symptoms of child diagnoses and percentage meeting criteria

		**All parents**		**Cohabiting parents**		**Non-cohabiting parents**	
	**Required # symptoms for diagn.**	**M (SD)**	**% children with diagn.**	**M (SD)**	**% children with diagn.**	**M (SD)**	**% children with diagn.**
ADHD	≥6	1.22 (.04)	1.9	1.12 (.05)	1.3	1.78 (.18)	6.5
ODD	≥4	.74 (.03)	1.8	.70 (.03)	1.5	.91 (.10)	4.2
CD	≥3	.32 (.06)	.7	.29 (.02)	.3	.50 (.06)	4.2
MDD	≥5	.59 (.06)	.3	.57 (.02)	.1	.76 (.08)	1.1
DyD	≥3	.51 (.02)	.2	.49 (.02)	.1	.65 (.07)	1.4
GAD	≥5	.43 (.02)	.6	.40 (.02)	.5	.63 (.07)	1.2
SAD	≥3	.31 (.02)	.3	.28 (.02)	.2	.50 (.06)	1.5

### Measurement model

The latent parent variable Self-preoccupation and impulsive aggressiveness was regressed on the symptoms of BPD, ASPD, and NPD. The latent child variable of Externalizing was regressed on the number of symptoms within each of the following main DSM-IV diagnoses: ADHD, ODD, and CD. The latent child variable of Internalizing was regressed on the number of symptoms within MDD, DyD, GAD, and SAD. All of the parameters for the latent parent variable and the two latent child variables were highly significant (p < .0001), for all parents as shown in Table 4.

**Table 4:** Measurement model: Estimates of latent parent and child variables

**Self-preoccupied & imp. aggressive parents by**	**All parents**				**Cohabiting parents**				**Non-cohabiting parents**			
	**B**	**SE B**	**β**	**R^2^**	**B**	**SE B**	**β**	**R^2^**	**B**	**SE B**	**β**	**R^2^**
BPD	1	0	.726****	.527	1	0	.732****	.536	1	0	.635****	.403
ASPD	.254	.047	.426****	.182	.262	.051	.434****	.188	.262	.051	.430****	.185
NPD	.312	.052	.421****	.177	.311	.053	.413****	.170	.311	.053	.426****	.181
**Child Externalizing by**												
ADHD	1	0	.537****	.288	1	0	.525****	.276	1	0	.537****	.288
Opposit. Defiant Dis.	.744	.130	.675****	.456	.745	.147	.647****	.419	.745	.147	.718****	.515
Conduct Disorder	.298	.050	.479****	.230	.317	.057	.484****	.234	.317	.057	.517****	.267
**Child Internalizing by**												
Depression	1	0	.962****	.923	1	0	.961****	.923	1	0	.958****	.919
Dysthymia	.921	.020	.972****	.949	.921	.021	.970****	.942	.921	.021	.983****	.967
GAD	.574	.034	.627****	.392	.579	.035	.619****	.384	.579	.035	.662****	.439
SAD	.111	.032	.141****	.029	.108	.031	.139****	.019	.108	.031	.124***	.015

***p < .001, ****p < .0001.

### Full SEM model

**Figure 3:**
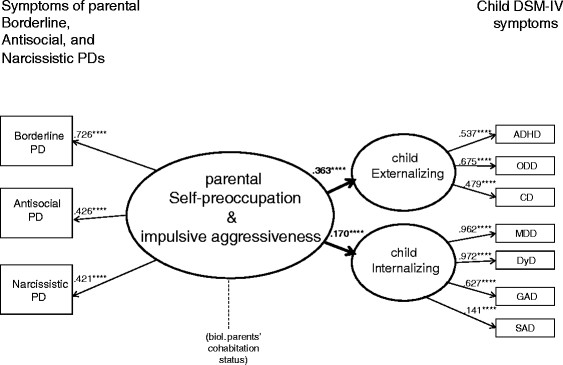
Full SEM model with significant pathways.

The main hypothesized model devised for testing was as follows: Does the latent parent variable Self-preoccupation and impulsive aggressiveness predict the two latent child Externalizing and Internalizing? The hypothesized model in Figure 2 offered good overall fit for the data and had the following fit indices: Chi-Square = 73.797, df = 24, CFI = .976, TLI = .963, and RMSEA = .047 (90% CI: .035-.060). All of the significant pathways for the measurement model and the full SEM for all of the parents are presented in Figure 3.

The results of the SEM are also presented in Table 5, with estimated parameters of how well parental Self-preoccupation and impulsive aggressiveness predicted the child symptoms of Externalizing and Internalizing.

**Table 5:** SEM estimates of the effect of the latent parent variable on latent child variables

**Child DSM-IV symptoms**	**Parents’ self-preoccupation & impulsive aggressiveness**											
	**All parents**				**Cohabiting parents**				**Non-cohabiting parents**			
	B	SE B	β	R^2^	B	SE B	β	R^2^	B	SE B	β	R^2^
Externalizing	.271	.065	.363****	.132	.240	.067	.344****	.118	.395	.177	.438****	.192
Internalizing	.099	.030	.169***	.029	.059	.030	.103*	.011	.298	.107	.437****	.191

*p < .05, ***p < .001, ****p < .0001.

As shown in Table 5, Self-preoccupation and impulsive aggressiveness explained 13.2% of the variance in the children’s Externalizing symptoms but only 2.9% of the Internalizing symptoms. However, when parents lived apart, the latent parent variable also predicted the children’s Internalizing symptoms, explaining 19.1% of the variance.

### Testing parental cohabitation as moderator

Additional models were tested to assess whether parental cohabitation status of biological parents moderated the prediction of the child variables. First a model was tested with cohabitation status as a grouping variable thereby obtaining parameter estimates of the two groups (cohabiting and non-cohabiting parents). This model is denoted as the free grouping model (with free parameters) and showed good fit of the data, with the following fit indices: Chi-square = 121.616, df = 60, CFI = .972, TLI = .96, and RMSEA = .048 (90% CI: .035-.060). The parameter estimates of cohabiting and non-cohabiting parents of this free grouping model are presented in Tables 4 and 5.

Subsequently, the free grouping model was repeated with parameter values that were fixed to be the same in the two groups (=no difference between cohabiting and non-cohabiting parents). This model was called the fixed grouping model and showed an acceptable, although somewhat larger Chi-square value (poorer fit): Chi-square 128.681, df = 62, CFI = .970, TLI = .965 and RMSEA .049 (90% CI: .037-.061). Significance of any difference in Chi-square values between the free and fixed grouping models was calculated using the Satorra-Bentler scaled chi-squared difference test [69]. This test yielded a value of 7.575, p = 0006, thereby indicating a significant moderation effect of parents’ cohabitation status on the association between the latent parent variable Self-preoccupation and impulsive aggressiveness and the parameters of the latent child variables Externalizing and Internalizing.

To determine which paths between the latent parental variable and the two latent child variables (Externalizing and Internalizing) were affected by the parents’ cohabitation status, the free grouping model was repeated with the parameter estimate of the parent variable on Externalizing being fixed to be the same in both groups, while the parameter of the other child outcome Internalizing was free. The Chi-square model fit of this partially fixed model could then be compared to the Chi-square model fit of the free grouping model to assess which was better. Significance of a difference in Chi-square between the two models was calculated with the Satorra-Bentler scaled Chi-square difference test and yielded a value of 1.174, p = .2786, NS. This indicated that the prediction of Self-preoccupation and impulsive aggressiveness on child Externalizing was the same for cohabiting and non-cohabiting parents.

Thereafter, the same procedure was repeated, but this time fixing parameters of ‘Internalizing’, and setting parameters of ‘Externalizing’ free to test whether cohabitation also was a moderator of the association of the parent variable and child ‘Internalizing’. The test of significance of difference in Chi-square between the two models yielded: 7.9763, p = .005. This indicated that the moderation variable of parental cohabitation status had a significant impact on the child outcome of Internalizing. The predictive value of parents’ Self-preoccupation and impulsive aggressiveness was significantly stronger for children’s Internalizing symptoms when parents were not living together.

## Discussion

This study examined the associations between parents’ self-reported symptoms of Borderline (BPD), Antisocial (ASPD), and Narcissistic Personality Disorders (NPD) and their preschoolers’ DSM-IV symptoms of behavioral and emotional diagnoses. Previous studies have documented that the children of parents with PD diagnoses are at risk for developing mental health problems themselves [6]. The current study showed for the first time that these findings extend to the population as well, with subclinical levels of BPD, ASPD and NPD predicting symptoms of behavioral and emotional diagnoses in children as young as preschool age. Importantly, the association between parental symptoms of BPD, ASPD and NPD and offspring symptoms of DSM-IV emotional disorders was considerably stronger when the parents were not living together.

Studies of individual risk factors leading to children’s mental health disorders seldom yield a high explanatory value (R^2^) of the variance in the children’s problems. This is naturally due to the multifactorial pathways and mediations that may lead to children’s behavioral and emotional difficulties. Hence, the finding of this study that in a large community sample, parents’ self-reported subclinical (not diagnosable, yet deviant) PD symptoms explained up to 19.1% of the variance of their children’s behavioral and emotional symptoms, while not alarming, nevertheless merits some concern.

### Generational transmission

This study can by no means disentangle the web of causal relations by which parents with symptoms of BPD, ASPD and NPD transmit behavioral and emotional problems in their preschool-aged children. Previous studies indicate a hypothesis that non-optimal parenting might mediate some of the relationship between parental PD symptoms and child problems [32-34]. In a representative community study of biological parents and their offspring conducted in New York, maladaptive parental behavior substantially mediated a significant association between parental and offspring psychiatric symptoms but only when there was a history of maladaptive parental behavior [32].

In the current inquiry, parental cohabitation status had a strong moderating effect on the association between parents’ Self-preoccupation and impulsive aggressiveness and child Externalizing and Internalizing symptoms. Children of non-cohabiting parents had a higher risk of child Externalizing symptoms compared with children of cohabiting parents, but they also showed a substantial increase in Internalizing symptoms when compared with children of parents living together. Externalizing and Internalizing symptoms are highly comorbid, especially in preschoolers [51]. Children of single parents with PD symptoms conceivably show more comorbidity than children living with both parents. A tentative interpretation may be that children living in a household with two parents among which one of the parents has BPD, ASPD or NPD symptoms may act out, protest, and be defiant, oppositional and restless. When a preschool child is left alone with such a parent, however, there are no corrective effects from the other parent that might have mitigated the effects from the parent with the PD symptoms. Chances that the child may also become scared and sad might increase. This interpretation could be in accord with research showing that older children of mothers with BPD report very low self-esteem [16]. However, we do not know the prevalence of PD symptoms in the other parent who did not participate in the study; therefore, this interpretation remains unconfirmed. Other explanatory factors than being left alone with a parent with PD symptoms cannot be ruled out.

The prevalence of BPD symptoms was somewhat higher among the non-cohabiting group of parents compared with parents living together (Table 2). Hence, one might infer that more BPD symptoms may account for some of the increase in child ‘Internalizing’ problems among non-cohabiting parents. Yet, when studying the parameter estimates for the latent variable ‘Self-preoccupation and impulsive aggressiveness,’ the symptoms of BPD among non-cohabiting parents did not contribute more to the variable than the BPD symptoms of cohabiting parents.

When interpreting the risk to children of having parents with BPD, ASPD and NPD symptoms, the bi-directionality of effects must be considered. Accordingly, a child with a difficult temperament challenges parents so that she or he elicits non-optimal parenting, which in turn aggravates the child’s behavior. It is likely that demanding children represent more of a provocation to parents who have irritable temperaments themselves and in addition may have difficulties putting aside their own wants and needs in favor of those of their children. The bi-directional escalation of non-optimal parent–child interactions may be further exacerbated and complicated by the parents’ inclination to attribute flaws and hostile intentions to those people who are close to them, including, conceivably, their children [5].

### Implications for practice

A common characteristic of persons with PDs is that they themselves most often do not consider their behavior to be problematic (i.e., the traits are ego-syntonic), yet their way of dealing with other people may represent a major stressor to persons who are close to them. Subsequently, parents with symptoms that are characteristic of BPD, ASPD and NPD may readily see faults and flaws in their children (and spouses) but rarely acknowledge that their own behavior or attitude contributes to any problems. Hence, the child psychiatric services that work with these parents must often develop a fine balancing act between maintaining a working alliance (or else one does not see the child or the parent again) and changing the dysfunctional parenting behavior that may sustain or aggravate child problems.

The findings from this study, i.e., the non-negligible risk to preschool-aged children due to the subclinical levels of parental PD symptoms, hold implications that are somewhat disquieting. The parents in this study represent the normal range of expected personality functioning, yet their children may suffer from the parents’ self-preoccupation and aggressive impulsivity. These parental features may be difficult for outsiders to identify because they most often manifest in close relationships. Furthermore, the parents themselves are seldom seeking help (except in cases of BPD symptoms of self-mutilation or suicidal behavior) due to the ego-syntonic nature of their symptoms. Nevertheless, this study indicates that providers who serve children in the community or in specialized facilities should have sufficient knowledge of PDs, specifically BPD, ASPD and NPD, to recognize them in parents. When in doubt, it would be opportune for child service providers to use self-report PD screening instruments to test parents for personality dysfunction. The providers could use the results to better balance and focus their work with the parents, thus preventing treatment dropout. Several researchers have advocated screening parents for psychopathology when their children receive a psychiatric evaluation [4]. The results of this study may justify including parental personality dysfunction in such a screening. Furthermore, community service providers and even teachers need to keep in mind that the preschool children of parents with BPD, ASPD and NPD symptoms may experience daily emotional turmoil, unpredictable parental outbursts and erratic behavior, which may affect the child’s self-esteem, whether or not symptom severity has manifested.

When service providers rely on parental accounts of child behavior, it might be germane to know to what extent the reports of parents with BPD, ASPD and NPD are based on distorted perceptions. Previous research has confirmed a depression-distortion hypothesis; high levels of maternal depression are related to over-reporting children’s behavior problems [70-72]. A major study has found that BPD mothers demonstrate thought problems in their relations [11], which may indicate a perception bias. However, only one known study to date has investigated parental personality disorders as possible distortions, and it found that personality disorders did not increase the discrepancies between teachers’ and parents’ reports in a low-risk sample [73]. Hence, a putative bias might only be evident in clinical samples.

### Strengths and limitations

The main strength of this study is the large community population, which is representative of the general population in Norway. Furthermore, the children in this study were not diagnosed by questionnaires but by an extensive psychiatric interview. Although the interview was conducted with the parents, it is an interviewer-based instrument and thus limits informant contamination.

An obvious limitation of the study is that we only obtained information about symptoms of PDs from the informant parent and not from the other parent. However, all but two of the participating informants had custody and were living with the child.

The parents’ DSM-IV symptoms of BPD, ASPD and NPD were derived from a self-reported questionnaire, and this method may represent a bias toward providing socially desirable responses when their children were the target of investigation. Parents may have underreported their own symptoms to avoid unwanted involvement from professionals. However, previous studies with the DIP-Questionnaire point to the opposite, i.e., the instrument has a somewhat better sensitivity than specificity, leading to a slight overreporting of symptoms compared with diagnostic interviews [60].

Future studies should investigate parenting as a possible mediating variable between parental symptoms of BPD, ASPD and NPD and their children’s behavioral and emotional difficulties.

## Conclusion

For the first time, subclinical levels of Borderline, Antisocial, and Narcissistic PD symptoms in parents have been documented to predict behavioral and emotional difficulties in their children as early as the preschool age. When parents were not cohabiting, the variance of the children’s emotional problems explained by parental symptoms increased more than six times. Child service providers need to have knowledge of those deviant personality traits in parents that may represent a possible peril to their children’s mental health, even when parental PD is not diagnosable.
